# Hinge-type FBG acceleration sensor based on double elastic plate

**DOI:** 10.1038/s41598-021-03628-7

**Published:** 2021-12-21

**Authors:** Zhongchao Qiu, Jinquan Zhang, Yuntian Teng, Zhitao Gao, Li Hong

**Affiliations:** 1 Institute of Geophysics, China Earthquake Administration, Beijing, 100081 China; 2 Hebei Key Laboratory of Earthquake Dynamics, Sanhe, 065201 China; 3 Institute of Disaster Prevention, Sanhe, 101601 China

**Keywords:** Natural hazards, Optics and photonics

## Abstract

It is critical for the health monitoring of large-scale structures such as bridge, railway and tunnel to acquire the medium-frequency and high-frequency vibration signals. To solve the problems of low sensitivity and poor transverse anti-interference of the medium-frequency and high-frequency fiber acceleration sensor, a hinge-type Fiber Bragg Grating(FBG) acceleration sensor based on double elastic plate has been proposed, and the hinge and elastic plate are used as elastomer to realize the miniaturization and transverse interference suppression of the sensor. The MATLAB and the ANSYS are used for theoretical analysis and optimization of sensor sensitivity and resonance frequency, structural static stress analysis and modal simulation analysis, while the test system is built to test the sensor performance. The results show that the resonance frequency of the sensor is 1300 Hz; the sensor has a flat sensitivity response in the middle-high frequency band of 200–800 Hz; the sensitivity is about 20 pm/g, and the fiber central wavelength drift and acceleration have good linearity and stability, while the transverse anti-interference is about 3.16%, which provides a new idea for monitoring of medium-frequency and high-frequency vibration signals in large-scale structures.

## Introduction

Mechanical vibration signals can be divided into low frequency, medium frequency and high frequency signals according to the frequency, and the high frequency vibration signals are signals with a frequency greater than 1 kHz. Usually in the range of high frequency, the acceleration of vibration is mainly measured, which represents the strength of impact force suffered by the vibration component. The magnitude of impact force is positively correlated with the acceleration value^1,2^. Therefore, the medium-frequency and high-frequency vibration performance will affect the structural health of large-scale infrastructure such as bridge, track and railway significantly^3–5^. The acceleration measurement, as an important technical means to monitor the medium-frequency and high-frequency vibration performance of engineering structures, can reflect the vibration situation of several large-scale structures^6,7^. At this stage, the electric sensor is the main instrument for acceleration measurement, and is characterized with low cost, small volume and relatively mature technology^8^; however, in the complex situations, it also has disadvantages of poor circuit stability, large signal noise and being is susceptible to electromagnetic interference^9,10^. Compared with the traditional electrical acceleration sensor, FBG acceleration sensor has the better sensitivity and linearity, better anti-electromagnetic interference and stability, easier distribution and measurement based on several parameters^11–13^. Therefore, FBG acceleration sensor is promising in the engineering application of acceleration detection.

Tianliang Li et al.^14^ put forward a diaphragm-type FBG acceleration sensor, which can improve the sensitivity and resonance frequency of the fiber based on the axial characteristics of the suspended fiber of diaphragm structure with a acceleration sensitivity of 20.189 pm/g and a resonance frequency of 600 Hz, as well as a cross-axis sensitivity less than 3.31% for low cross sensitivity. However, it has low linearity of 0.764% and bandwidth of 10–200 Hz, which cannot meet the needs of medium-frequency and high-frequency engineering structure. Khan M M et al. ^15^ proposed a FBG acceleration sensor based on cantilever beam structure with L-shaped non-even cross section, which can realize the temperature self-compensation with double FBG. When the vibration frequency is lower than 50 Hz, the sensor sensitivity is 40 pm/g, and when the vibration frequency is higher than 150 Hz, the sensor sensitivity is 306 pm/g. Casas-Ramos et al.^16^ put forward a new cantilever-type FBG vibration sensor with a resonance frequency of 227.3 Hz, operation bandwidth of 10–210 Hz, a resolution of 0.006 g, a linearity and relative sensitivity error of 1.9% and ± 4.4% respectively. Du Wang et al.^17^ proposed a double-mass block acceleration sensor based on FBG. The proposed sensor is composed of FBG, beam and double mass block, and the FBG is fixed in the middle of the upper surface of the dual mass block by epoxy glue. The experimental results show that the natural frequency of the sensor is 1000 Hz, the sensitivity is 26.7 pm/g when the frequency is 250 Hz, and the lateral anti-interference degree is less than 6%. Wu Hao et al.^18^ put forward an acceleration sensor with an isosceles triangular cantilever and mass block structure, which has high natural frequency and high sensitivity. One section of the fiber grating is pasted on the cantilever and serves as an accelerometer; the other is used as a reference grating. The first-order natural frequency of the accelerometer is 8356 Hz, and its sensitivity is 0.46 pm/g. The sensor has the advantages of high frequency and sensitivity, and thus can be applied to the rail transit bearing systems. A general characteristic of the FBG accelerometer sensor is that its sensitivity and resonant frequency contradict each other. FBG accelerometer has low sensitivity in middle and high frequency, and has problems of transverse interference and linearity.

This paper presents a hinge-type FBG acceleration sensor based on double elastic plate, which can realize the miniaturization and transverse interference suppression of the sensor with the hinge and the elastic plate as elastomer. The MATLAB and the ANSYS are used for the theoretical analysis and optimization of sensitivity and resonance frequency, the static stress analysis of the structure and modal simulation of sensor structure. The physical sensor has been made based on the simulation results and subject to the experimental measurement in performance.

## Sensor design

### Sensor structure

**Figure 1:**
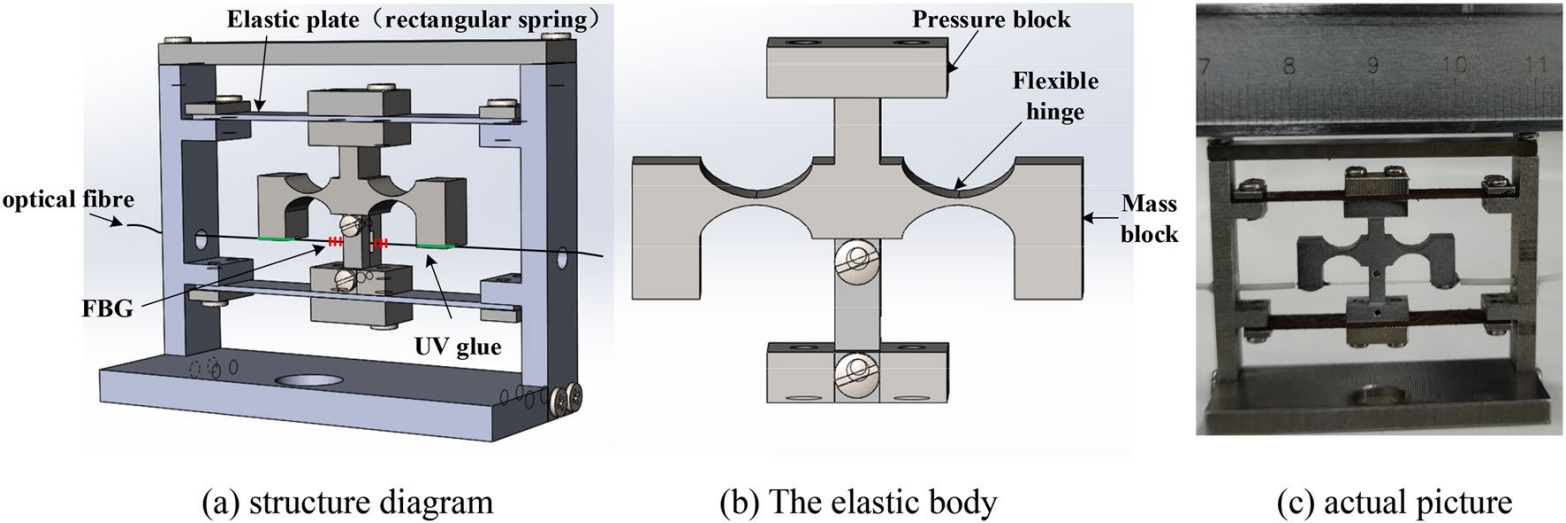
Sensor structure model.

The FBG sensor consists of the upper cover, pressure block, rectangular spring, pressure pad, column, elastic body and base as shown in Fig. 1, of which the column, upper cover and base are connected through screws, and the screws run through the gasket, rectangular spring and threaded hole on the column to fix the rectangular spring firmly. With the rectangular springs fixed in the upper and lower ends, the sensor structure can be more stable, and the vibration interference in the non-sensitive direction can be reduced. The elastic body is integral, and there is square hole for space required by fiber vibration reserved at the lower end of base column, and the mass blocks at both ends are connected with each other through the flexible elliptical hinge and middle base. There are through holes arranged in the relevant positions of the housing at both ends of the elastic body. FBG is placed in the groove of the mass block upon a pre-stressing force is imposed to a certain extent, while two ends are fixed through UV glue. When prestressing, one end of the fiber is hung with a weight and the other end is connected with the FBG interrogator. By changing the mass of the weight, the center wavelength of the fiber is increased by about 1 nm. At this time, the fiber grating is stretched straight and not easy to be broken in the subsequent vibration test.

In the measurement, the sensor is placed horizontally through the circular hole of the base. When the measured object vibrates, the pressing block and the elastic body deform the elastic plate under the action of inertia, and the deformation of the elastic plate is transmitted to the mass block in the elastic body. The FBG is connected with the mass block and the base under two-point packaging technique, and the FBG is suspended between them. In case of vibration of the mass block, two mass blocks will rotate slightly in an opposite direction around the hinge, and the FBG is stretched or compressed, while its central wavelength will drift. The relationship between the central wavelength of the strain is expressed as:

1 $$\frac{\Delta \lambda }{\lambda } = (1 \, - \, P_{e} )\varepsilon + \, (\alpha_{\Lambda } + \alpha_{n} )\Delta T$$

where $\lambda$ is the central wavelength of FBG; $P_{e}$ is the photoelastic coefficient; $\alpha_{\Lambda }$ is the coefficient of thermal expansion; $\alpha_{n}$ is the thermal optical coefficient.

As FBG is sensitive to both strain and temperature, the temperature variation should be controlled within a small range in the experiment. In the sensor physical test, the whole testing process is controlled at room temperature of 27℃. There is only a temporary temperature rise when UV glue is cured by UV lamp irradiation. The sensor is cooled for a short time and then tested to avoid the influence of temperature on the optical fiber.

### Sensor sensitivity analysis

**Figure 2:**
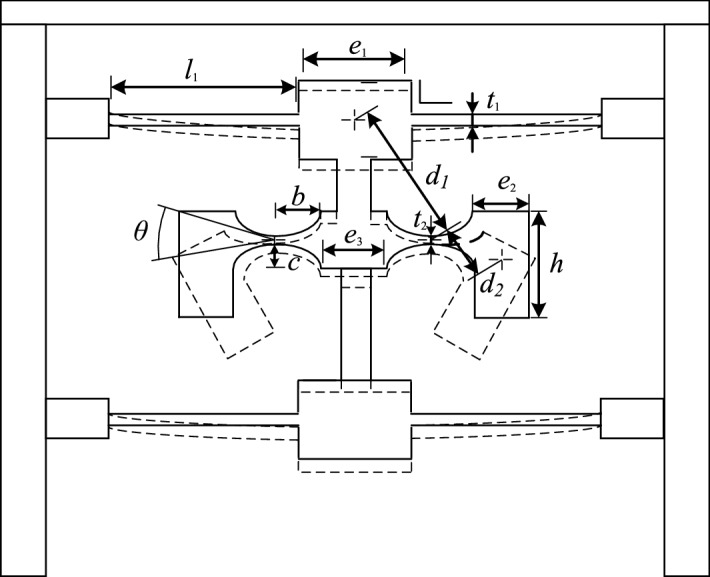
Mechanical model of sensor.

The mechanical model of the sensor is shown in Fig. 2. When the incentive acceleration is along the vertical direction of the sensor, two columns can be considered as a rigid body, since the deformation of hinge in the elastic body is greater than that of the column end, and with the flexible hinge replaced by the ideal hinge, the torque balance can be realized for the whole system under the inertia effect, and the balance equation can be expressed as:

2 $$2m_{1} ad_{1} + 2m_{2} ad_{2} - 2k_{1} \Delta l_{1} - k_{f} \Delta l_{f} \frac{h}{2} - 2k_{2} \theta = 0$$

where$m_{1}$ and $m_{2}$ are the mass of the pressure block and mass block; $d_{1}$ and $d_{2}$ are the distance of the pressure block and the center of mass block from the hinge center; $\Delta l_{1}$ is the displacement of the elastic plate; $\Delta l_{f}$ is the stretching distance of the fiber; $h$ is the height of the mass block; $k_{f}$, $k_{1}$ and $k_{2}$ represent the elastic coefficient of the fiber, the elastic coefficient of the elastic plate and the hinge rotation stiffness respectively, and $\theta$ is the central hinge rotation angle. The semi-major axis of elliptical hinge is $b$. Since low hinge rotation angle, the following can be obtained:

3 $$\theta = \frac{{2\Delta l_{f} }}{h}$$

When the length of the pressure block is $e_{1}$; that of the mass block is $e_{2}$, and that of the elastic base is $e_{3}$, it can be obtained that $d_{{1}} = b + e_{1} /2$, $d_{2} = b + e_{2} /2$, $l_{f} = 4b + e_{3}$ and $\Delta l_{1} = (2b + e_{2} )\theta$.

The elastic coefficient of the fiber $k_{f}$ is

4 $$k_{f} = \frac{{A_{f} E_{f} }}{{l_{f} }}$$

where, $A_{f}$ is the cross sectional area of the fiber, and $E_{f}$ is the elastic modulus of the grating. With the rectangular elastic plate used, the transverse vibration interference can be reduced. The elastic coefficient of the elastic plate can be expressed as:

5 $$k_{1} = \frac{{E_{1} b_{1} t_{1}^{3} }}{{6l_{1}^{3} }}$$

where, $E_{1}$ is the elastic modulus of the elastic plate with equal strength; $b_{1}$ is the bottom width of the elastic plate with equal strength; $t_{1}$ is the thickness of the elastic plate with equal strength; $l_{1}$ is the length of the elastic plate with equal strength.

The rotational stiffness is critical for the sensor, and based on the theoretical formula of hinge stiffness, the rotational stiffness of the hinge can be expressed as:

6 $$k_{2} = \frac{{Ewt_{2}^{3} }}{24bu}$$

where

7 $$u = \frac{{12s^{3} + 14s^{2} + 6s + 1}}{{(2s + 1)^{2} (4s + 1)^{2} }} + \frac{6s(2s + 1)}{{(4s + 1)^{5/2} }}\arctan \frac{1}{{\sqrt {4s + 1} }} + \frac{{6s(8s^{3} + 12s^{2} + 6s + 1)}}{{(2s + 1)^{2} (4s + 1)^{5/2} }}\arctan \frac{2s}{{\sqrt {4s + 1} }}$$

where, $E$ is the elastic modulus of the material; $w$ is the thickness of the hinge; $c$ is the semi-minor axis of the elliptical hinge; $t_{2}$ is the minimum thickness between hinges; the sensor sensitivity is the ratio of the central wavelength variation of the FBG to the acceleration, and the following can be obtained from the Eqs. (1) and (2)

8 $$S = \frac{\Delta \lambda }{a} = \frac{{(1 - P_{e} )\lambda_{B} }}{{l_{f} }}\frac{{4(m_{1} d_{1} + m_{2} d_{2} )h}}{{8k_{1} (2b + e_{2} ) + 4k_{2} + k_{f} h}}$$

where $P_{e}$ is the elastic-optic coefficient; $\lambda_{B}$ is the central wavelength of the grating; $\varepsilon_{f}$ is the fiber strain; the sensitivity below is the peak-peak sensitivity $2S$.

### Sensor resonance frequency analysis

The resonance frequency $f$ is another important parameter of the acceleration sensor, and it is closely related to the available bandwidth of the sensor. In general, the higher resonance frequency leads to wider available bandwidth of the sensor. It is assumed that the rotational inertia of the mass block around the hinge center is $J$. The potential energy of strain of the fiber can be:

9 $$J\mathop \theta \limits^{..} + [2k_{1} (e_{2} + 2b)^{2} + k_{f} (h/2)^{2} + 2k_{2} ]\theta = 0$$

After the Eq. (8) is substituted into the kinetic equation, the resonance frequency of the whole system is:

10 $$f = \frac{1}{2\pi }\sqrt {\frac{{{2}k_{1} (e_{2} + 2b)^{2} + k_{f} (h/2)^{2} + {2}k_{2} }}{J}}$$

where the rotational inertia is

11 $$J = m_{{1}} \frac{{e_{{2}}^{2} + h^{2} }}{12} + m_{{1}} d_{1}^{{^{2} }}$$

## Sensor structure simulation analysis

### Influence of structural parameters on the sensor

According to Eqs. (8) and (10), the elastic coefficient of the elastic plate and the rotational stiffness of the hinge increase, the sensor sensitivity decreases, and the resonant frequency increases. Among them, the key parameters of hinge including the minimum thickness between hinges $t_{2}$, the semi-minor axis of the elliptical hinge $c$, the semi-major axis of the elliptical hinge $b$, the key parameter of the elastic plate including the bottom width of the elastic plate $b_{1}$ and the thickness of the elastic plate $t_{1}$, and these parameters have larger adjustment space in the sensor production, so to analyze the five parameters using MATLAB. Since the minimum thickness between the hinges is small, only three fixed values are selected in the analysis, considering that the machining process is very easy to produce errors.The sensor is made of 65 manganese, the elastic modulus is 210 GPa; the density is 7850 kg/m^3^; the sensor width is 7 mm; the elastic modulus of the fiber is 72 GPa; the effective elastic-optic coefficient is 0.22; the central wavelength of the FBG is 1550 nm, and the length is 5 mm.

From Eqs. (8) and (10), it can be known that some key parameters of the sensor, including the minimum thickness between hinges $t_{2}$, the semi-minor axis of the elliptical hinge $c$, the semi-major axis of the elliptical hinge $b$, the bottom width of the elastic plate $b_{1}$ and the thickness of the elastic plate $t_{1}$, all can influence the sensitivity and resonance frequency significantly, and these parameters can be adjusted greatly in the fabrication of the sensor. Upon analysis on five parameters with MATLAB under the sensor material of 65Mn, the elastic modulus is 210 GPa; the density is 7850 kg/m^3^; the sensor width is 7 mm; the elastic modulus of the fiber is 72 GPa; the effective elastic-optic coefficient is 0.22; the central wavelength of the FBG is 1550 nm, and the length is 5 mm.

**Figure 3:**
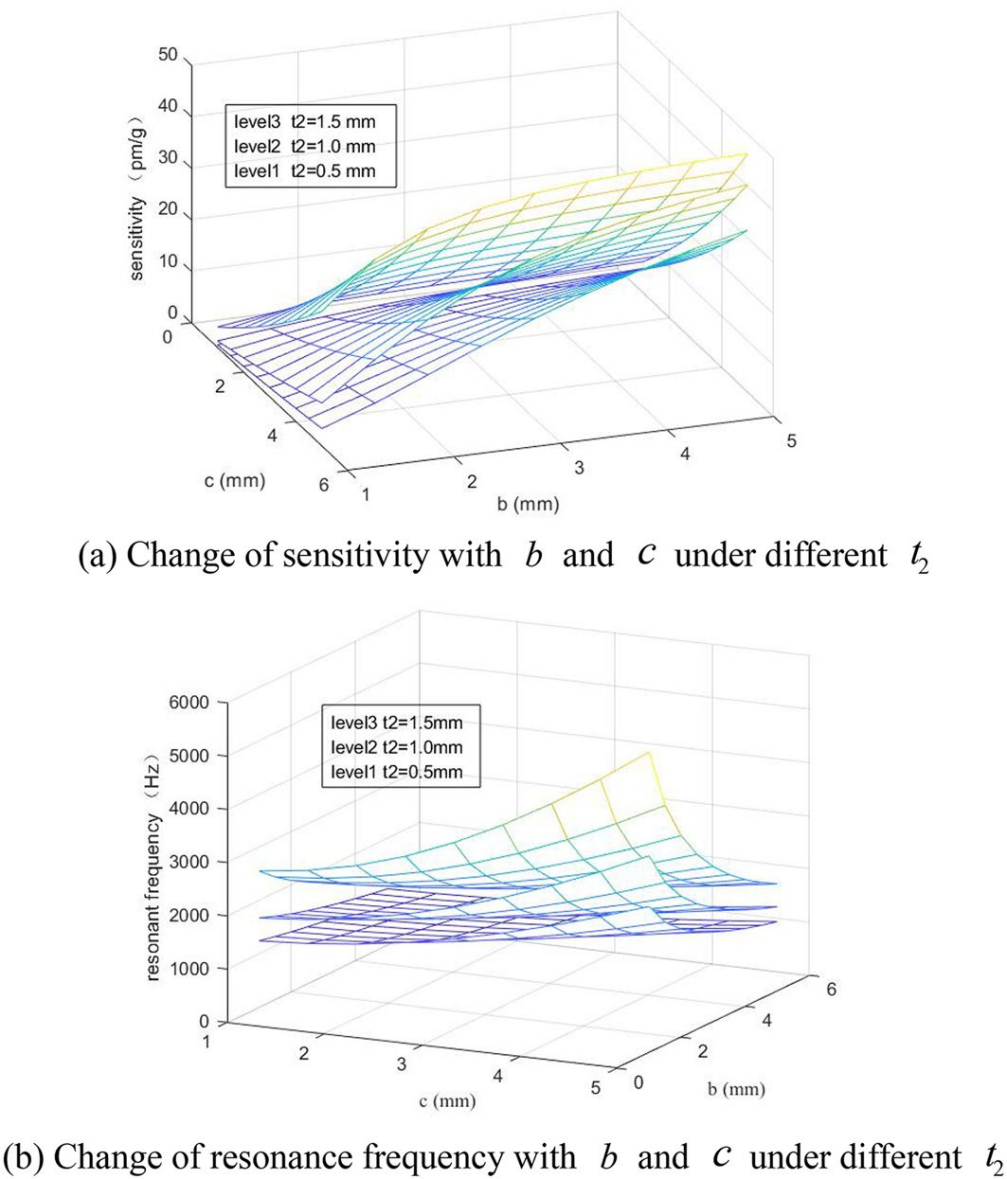
Influence of parameters $b$ and $c$ on sensor performance.

The influence of $b$ and $c$ on the sensor sensitivity and resonance frequency when $t_{2}$ = 0.5 mm, 1.0 mm and 1.5 mm has been discussed in the first group, and after it is assumed that $b_{1}$ = 5 mm, $t_{1}$ = 0.5 mm, 1 mm ≤ $b$ ≤ 5 mm and 1 mm ≤ $c$ ≤ 5 mm, the sensor sensitivity is obtained as shown in Fig. 3a and the resonance frequency is shown in Fig. 3b.

From Fig. 3, it can be known that the minimum thickness of the hinge $t_{2}$, the semi-major axis of the elliptical hinge $b$ and the semi-minor axis of the elliptical hinge $c$ can influence the sensor sensitivity and resonance frequency significantly. When the sensitivity $b$ is increased, the resonance frequency will be decreased, and when $c$ is increased, the sensitivity and resonance frequency are increased. When $t_{2}$ is increased, the resonance frequency is increased greatly. To meet the measurement needs of the sensor, the resonance frequency is within 1600 Hz, and the sensitivity is greater than 12 pm/g, while it is required that $c$ < 3 mm and $b$ > 2 mm.

**Figure 4:**
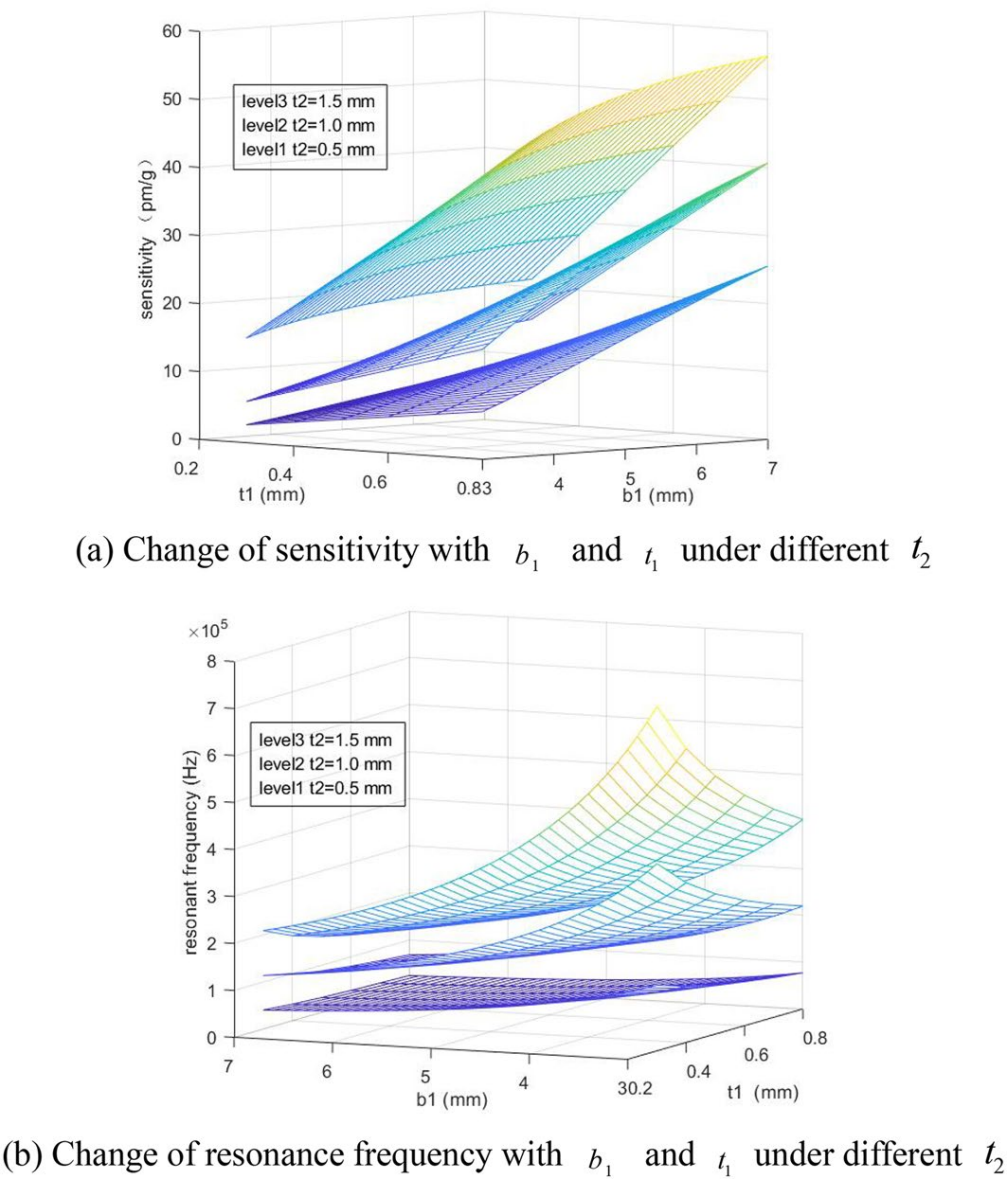
Influence of parameters $b_{1}$ and $t_{1}$ on sensor performance.

The influence of $b_{1}$ and $t_{1}$ on the sensor sensitivity and resonance frequency when $t_{2}$ = 0.5 mm, 1.0 mm and 1.5 mm has been discussed in the second group, and after it is assumed that $c$ = 2.5 mm, $b$ = 2.5 mm, 3 mm ≤ $b_{1}$ ≤ 7 mm and 0.3 mm ≤ $t_{1}$ ≤ 0.8 mm, the sensor sensitivity is obtained as shown in Fig. 4a and the resonance frequency is shown in Fig. 4b.

From Fig. 4, it can be known that the minimum thickness of the hinge $t$, the bottom width of the elastic plate $b_{1}$ and the thickness of the elastic plate $t_{1}$ can influence the sensor sensitivity and resonance frequency significantly. When $b_{1}$ and $t_{1}$ is increased, the sensitivity will be increased, and the resonance frequency will be decreased. When $t_{2}$ is increased, the resonance frequency will be increased greatly. To meet the measurement needs of the sensor, the resonance frequency is within 1600 Hz, and the sensitivity is greater than 12 pm/g, while it is required that $t_{2}$ = 1.0 mm, $b_{1}$ > 4 mm and $t_{1}$ > 0.4 mm.

### ANSYS simulation analysis

From the analysis on structural parameters, it can be known that the sensor sensitivity and resonance frequency will be influenced significantly when the semi-major axis of the elliptical hinge $b$, the semi-minor axis of the elliptical hinge $c$, the bottom width of the elastic plate $b_{1}$ and the thickness of the elastic plate $t_{1}$ change little. Based on the needs of engineering application, it should be ensured that the resonance frequency is lower than 1600 Hz, and the sensitivity is higher than 12 pm/g. At the same time, since the size and weight of the sensor, it is required that $t$ = 1.0 mm, $b$ = 2.5 mm, $c$ = 3 mm, $b_{1}$ = 5.0 mm and $t_{1}$ = 0.5 mm. ANSYS is used for static stress and modal simulation of the sensor parameters. Table 1 presents the sensor parameters.

**Table 1:** Parameters of FBG Acceleration Sensor.

Name	Description	Length(mm)
*t*	Minimum thickness between hinges	1.0
*c*	Semi-short axis of the elliptical hinge	3.0
*b*	Semi-major axis of the elliptical hinge	2.5
*b*_1_	Width of the elastic plate	5.0
*t*_1_	Thickness of the elastic plate	0.5

**Figure 5:**
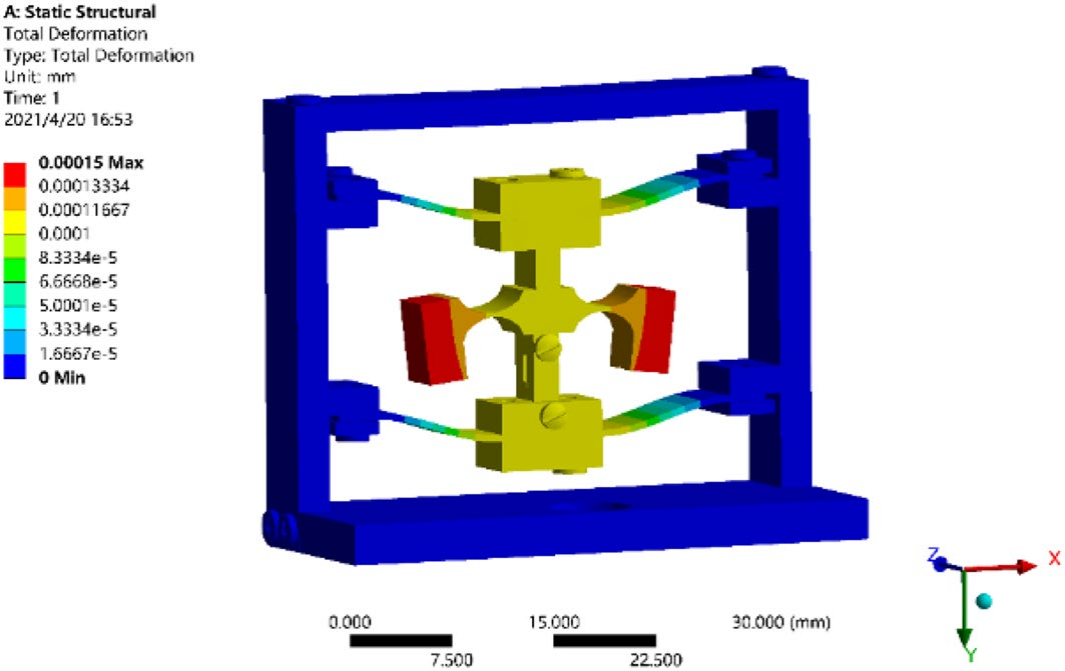
Static stress analysis of sensor structure.

The sensor is modeled with the structural parameters obtained by the optimization as above, and it should be imported into ANSYS for simulation analysis. Hexahedral cells are generated by mesh division with sweep, the size of hinge cells is 0.1 mm, and the size of the rest cells is 0.5 mm. The fixed constraint is applied to the sensor model, while 1 g external acceleration load is applied to the whole sensor to obtain the equivalent strain cloud of the model as shown in Fig. 5. From Fig. 5, it can be known that there is the maximum deformation from the free end of the sensor, and the deformation is decreased to the fixed end. With the maximum deformation of the free end of 0.15, it indicates that the response of external vibration signal can be realized by the sensor structure, and the deformation has no influence on the physical performance of the fiber, and the stability of the sensor can be guaranteed.

**Figure 6:**
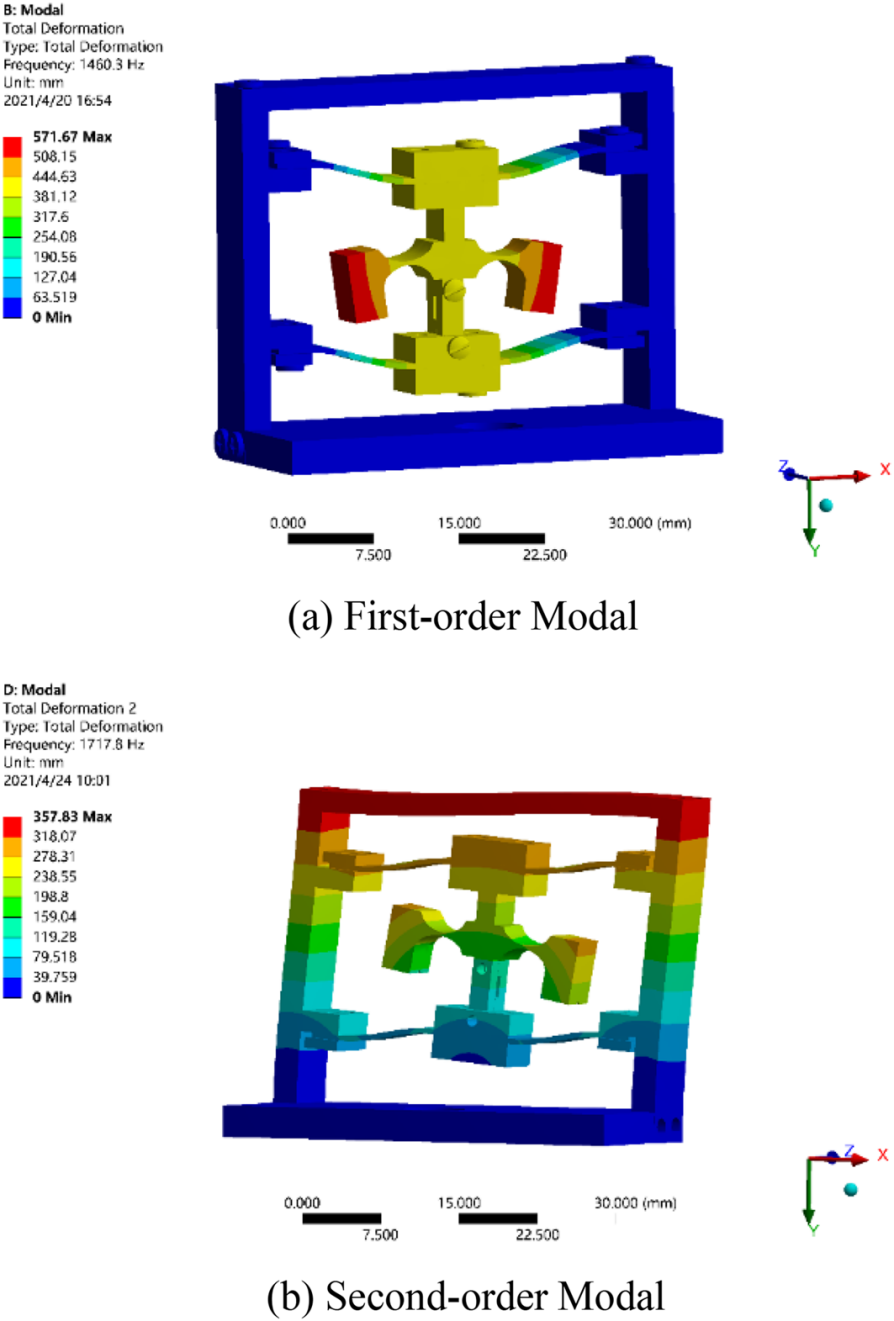
Modal analysis on sensor structure.

The modal analysis is made on the sensor model based on the results of static stress analysis, and the fixed constraint is applied to the base, while the mesh generation is built for the whole model. Upon the modal analysis on the model, the modal frequencies of the first four orders of the sensor can be obtained, and are 1460.3 Hz, 1717.8 Hz, 2251.5 Hz, and 2675.0 Hz, respectively. The first-order and second-order modals as shown in Fig. 6. From Fig. 6, it can be seen that the resonance frequency of each order of the sensor is different greatly, which shows that the structural sensor has small cross coupling and this can reduce the cross interference.

## Sensor test and analysis

**Figure 7:**
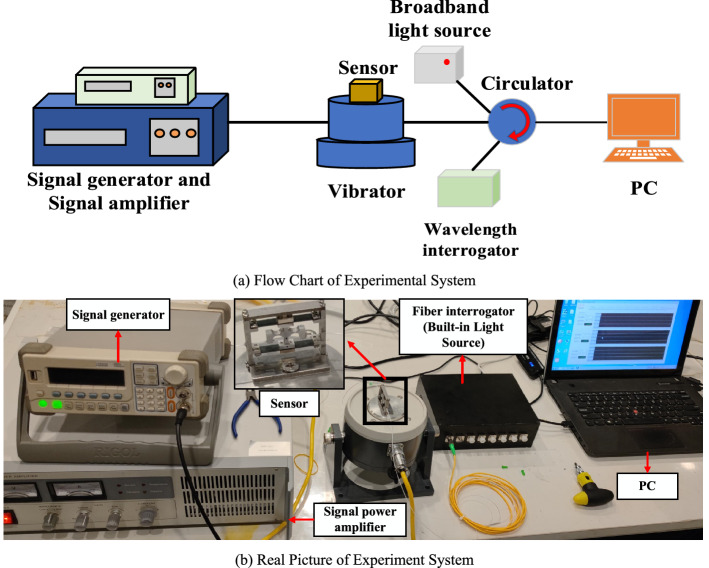
Sensor vibration test system.

The sensor test system mainly includes the function signal generator, signal amplifier, vibrator, FGB interrogator and computer as shown in Fig. 7. The function signal generator is of RIGOL series DG1022 model with a sampling frequency of 1 GSa/a, 14 quasi-waveform functions and rich standard configuration interfaces, which can support the users to remotely control the instrument and transmit the data of USB interface through Web. The signal amplifier is of MWY-TZQ50 model from Beijing Weiyun Technology Co. Ltd. with a frequency response range of 1–15,000 Hz and a signal-to-noise ratio higher than 75 dB. If it is used with a function signal generator, the function signal can be amplified. The FBG interrogator is of MWY-FBG-CS800 model from Beijing Weiyun Technology Co. Ltd. a wavelength range of 1527–1568 nm, wavelength resolution of 0.1 pm, dynamic range of 40 dB and sampling frequency up to 1 kHz, and there is a built-in laser light source, and the light wave emitted is transmitted to the acceleration sensor on the vibrator system through the fiber. Meanwhile, the FBG interrogator can receive the reflection spectrum of FBG, and can finish the spectrum analysis and data acquisition in it, and finally send the data acquired to the computer. According to the FBG interrogator's requirements, FBG adopts 1550 nm central wavelength, reflectivity ≧90%, edge touch suppression ratio ≧15 dB, and grating length 10 mm. FBG is irradiated by ultraviolet laser and made by Beijing Weiyun Technology Co. Ltd. With the above equipment, the FBG acceleration sensor test system can be built. The amplitude-frequency characteristics, sensitivity coefficient, stability and transverse anti-interference capability of the sensor can be tested in performance with the system, and the performance parameters of the sensor are obtained.

### Amplitude-frequency characteristic test

**Figure 8:**
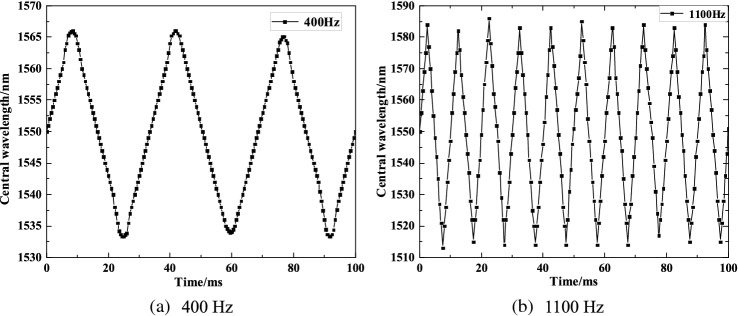
Frequency response of the sensor at 400 Hz and 1100 Hz.

**Figure 9:**
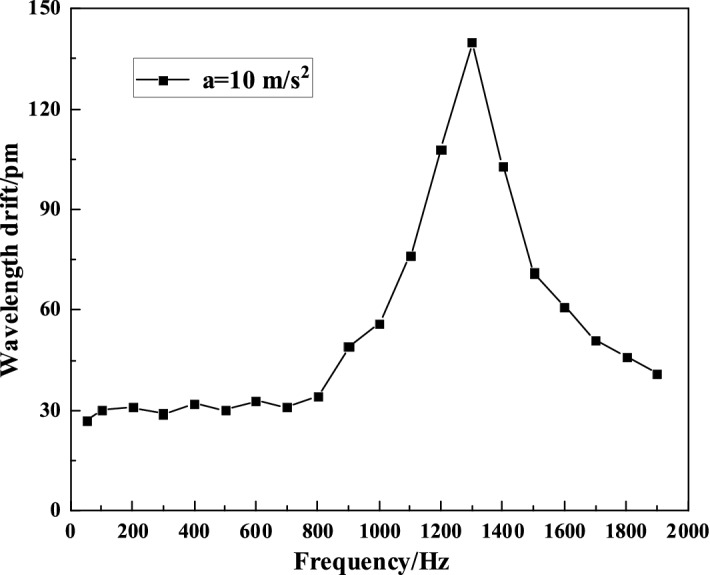
Amplitude-frequency response characteristics of sensor.

The frequency band range of the sensor depends on the frequency–response curve, so the developed sensor must be calibrated dynamically. The acceleration 10 m/s^2^ should be input in the amplitude-frequency test in the sensor as a constant acceleration value. Firstly, the wavelength variation of the sensor under FBG of 100 Hz is measured, and then the wavelength variations should be recorded every progressive increase of 100 Hz as 1 step from 100 Hz. The time-domain response curves of the sensor at 400 Hz and 1100 Hz are shown in Fig. 8, while the amplitude-frequency characteristic curve of the sensor is shown in Fig. 9.

From Fig. 8, it can be known that the sensor has the good time-domain response characteristics. From Fig. 9, it can be seen that the sensor is up to the maximum wavelength variation at about 1300 Hz, which means that the resonance frequency of the sensor is approximately 1300 Hz. There is a relatively good flatness at 200–800 Hz, and there is a certain error between the actual measured resonance frequency of the sensor and the simulation result upon the theoretical analysis. This is because: (1) the acceleration sensor is small in size and the hinge structure is partially thin, and there is a certain error for insufficient accuracy of processing; (2) no pre-stressing force is considered in the finite element method, and the prestress applied to FBG will affect the resonant frequency of the sensor; (3)the insufficient stability of the UV glue makes the strain generated by the elastomer not completely transferred to the FBG, that is, the strain transfer coefficient between the FBG and the elastic element is not equal to 1, at the same time, if the amount of UV glue on both sides of the groove is not symmetrical, it will make the two sides of the hinge rotation Angle is inconsistent, affecting the resonant frequency of the sensor; (4) the vibrator and demodulating system accuracy will lead to a certain error of the test data.

### Sensitivity coefficient test

**Figure 10:**
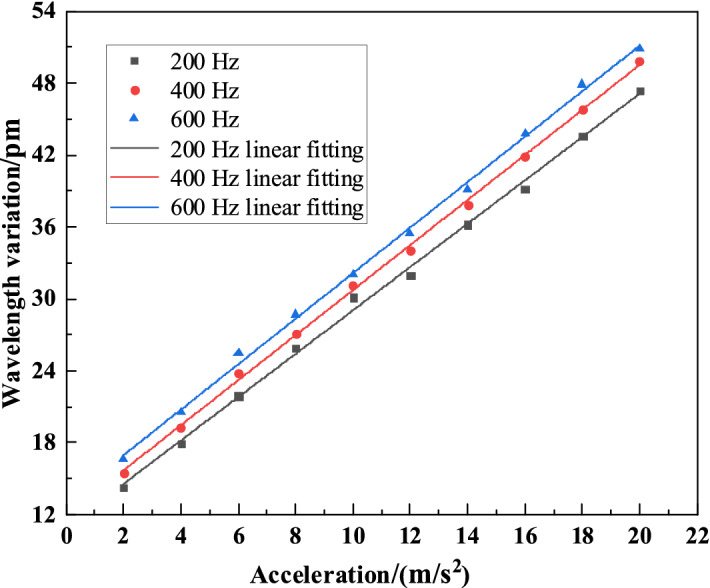
Sensor sensitivity calibration curve.

To obtain the sensitivity characteristics of the sensor, 200 Hz, 400 Hz and 600 Hz are used as test frequencies for sensor sensitivity calibration, and the acceleration step of the vibrator is changed to 2 m/s^2^ and is increased from 2 to 20 m/s^2^, while FBG change data at the central wavelength of different accelerations is recorded, and the central wavelength variation curve is drawn as Fig. 10.

From Fig. 10, it can be known that the sensor sensitivity is 19.25 pm/g under the frequency of 200 Hz, and the coefficient of fitting determination is R^2^ = 0.9977. When the frequency is 400 Hz, the sensor sensitivity is 19.55 pm/g, and the coefficient of fitting determination is R^2^ = 0.9990. When the frequency is 600 Hz, the sensitivity is 20.3 pm/g, and the coefficient of fitting determination is R^2^ = 0.9981. From this, it can be known that the sensor has good linearity.

In addition, dynamic range DR is also an important parameter to estimate the sensing characteristics of FBG vibration sensor. It generally reflects the ability of the sensor to receive strong and weak signals. The dynamic range of the sensor is directly related to its detectable acceleration range, which depends on the ratio of the maximum acceleration value to the minimum acceleration value. According to the relationship between vibration acceleration and wavelength, it can be expressed as:

12 $$D_{R} = 20\lg (\frac{{\lambda_{\max } }}{{\lambda_{\min } }})$$

The maximum wavelength shift value $\lambda_{\max }$ is mainly restricted by the elastic deformation range of the elastic main body of the sensor and the prestress of the grating, which is 1690 pm as shown in Fig. 9. The minimum wavelength drift value $\lambda_{\min }$ is mainly determined by the resolution of the FBG interrogator, which is 0.1 pm. The dynamic range of the sensor can reach 84 dB.

### Stability test

To test the sensor stability, the output frequency of the vibrator is adjusted to be 400 Hz and 600 Hz respectively, and the accelerations are 6 m/s^2^, 10 m/s^2^ and 14 m/s^2^. The output of the sensor is recorded every 15 min. In this test, the relative standard deviation $RSD$ is used to represent the repeatability error of the sensor, and it is expressed as:

13 $$RSD = \frac{SD}{{\overline{x}}} \times {{100\% = }}\frac{{\sqrt {\frac{{\sum\nolimits_{i = 1}^{n} {(x_{i} - \overline{x})^{2} } }}{n - 1}} }}{{\overline{x}}} \times {{100\% }}$$

where, $SD$ is the standard deviation, and $\overline{x}$ is the corresponding mean value.

**Figure 11:**
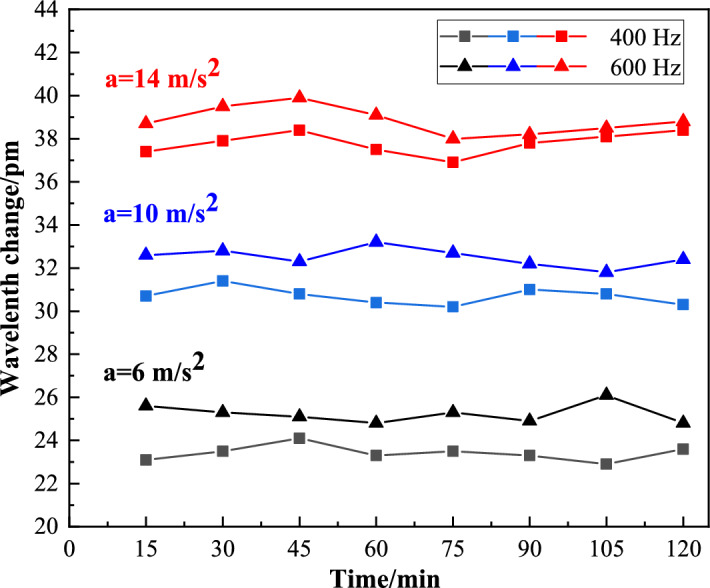
Sensor stability.

The test results are shown in Fig. 11. When the frequency is 400 Hz, the relative standard deviations of FBG central wavelength drift corresponding to 6 m/s^2^, 10 m/s^2^ and 14 m/s^2^ are 1.53%, 1.29% and 1.45%, respectively. When the frequency is 600 Hz, the relative standard deviations of the corresponding FBG central wavelength drift are 1.77%, 1.30%, and 1.65%, respectively. Therefore, we can know that there is small repeatability error of the sensor and good stability. In 2 h, the wavelength changes measured intermittently every 15 min are basically the same. Considering the errors caused by shaking table and optical fiber demodulation instrument, the calculated repeatability error of the sensor is acceptable, indicating that the sensor has good stability.

### Transverse anti-interference capability

**Figure 12:**
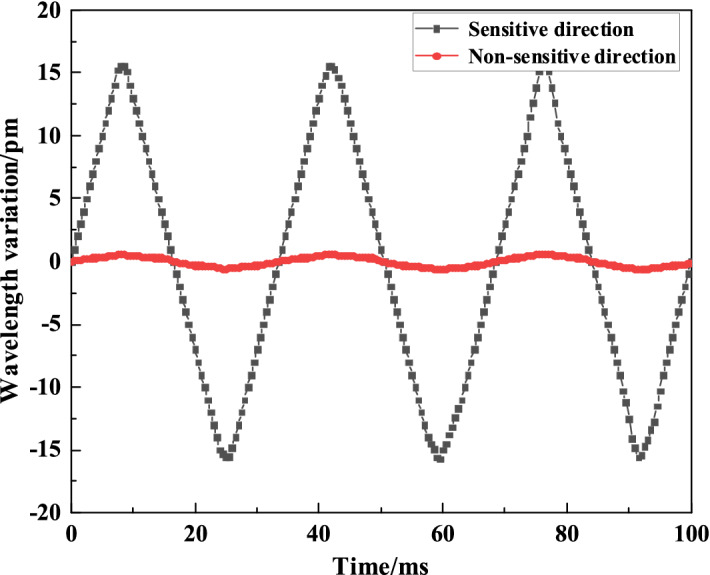
Transverse characteristics of the sensor.

As FBG acceleration sensor has a single degree of freedom, its transverse anti-interference capability is one of important indexes of the sensor. The sensor is fixed on the vibrator, and the frequency and the acceleration are adjusted to be 400 Hz and 10 m/s^2^ respectively. In the same vibration environment, the drift of FBG central wavelength under the transverse vibration and longitudinal vibration of the sensor is recorded, while the transverse anti-interference characteristics of the sensor are shown in Fig. 12.

From Fig. 12, it can be seen that the drift in a sensitive direction of the sensor is approximately 31.6 pm, and that in a non-sensitive direction will not be more than 1.0 pm, while the wavelength drift in a non-sensitive direction of sensor is only 3.16% of that in a sensitive direction. Experiments show that the proposed dual-elastic plate structure can effectively reduce the lateral rotation of the sensor element under the premise of good sensitivity, so that the sensor has strong anti-lateral interference characteristics.

## Conclusion

To solve the problems of low sensitivity and poor transverse anti-interference of the medium-frequency and high-frequency fiber acceleration sensor, a hinge-type FBG acceleration sensor based on double elastic plate has been proposed. The MATLAB is used for theoretical analysis and optimization of sensor sensitivity and resonance frequency, as well as the analysis and optimization of hinge thickness, hinge radius, mass block size and other structural parameters of the sensor, and the ANSYS is utilized for the structural static stress analysis and modal simulation analysis, while the test system is built to test the sensor performance. The results show that the resonance frequency of the sensor is 1300 Hz; the sensor has a flat sensitivity response in the middle-high frequency band of 200–800 Hz; the sensitivity is appropriately 20 pm/g, and the fiber central wavelength drift and acceleration have good linearity and stability, while the transverse anti-interference is appropriately 3.16%, which provides a new idea for monitoring of medium-frequency and high-frequency vibration signals in large-scale structures.

## References

[1] Shipley TS (2018). Effects of High Frequency Acceleration Device on Aligner Treatment—A Pilot Study. Dent. J..

[2] Liu C, Wang W, Qiao L (2020). A High-Frequency Acceleration Sensor for Monitoring Sloshing Response of Ships[C]//International Conference in Communications, Signal Processing, and Systems.

[3] Fang W, Bruni S (2019). A time domain model for the study of high frequency 3D wheelset–track interaction with non-Hertzian contact. Multibody Sys.Dyn..

[4] Loktev, A. A., Korolev, V. V. & Shishkina, I. V. High Frequency vibrations in the elements of the rolling stock on the railway bridges. In *IOP Conference Series: Materials Science and Engineering*, Vol. 463(3), 032019 (2018).

[5] Chan H, Masserey B, Fromme P (2015). High frequency guided ultrasonic waves for hidden fatigue crack growth monitoring in multi-layer model aerospace structures. Smart Mater. Struct..

[6] Zhou C, Tong X, Mao Y (2019). Study on a high-temperature optical fiber F-P acceleration sensing system based on MEMS. Opt. Lasers Eng..

[7] Hong L, Wang JH, Yao ZJ (2019). Sensitivity improvement of a new structure crack meter with angular adjustment. Meas. Control.

[8] Ghoddus H, Kordrostami Z, Amiri P (2019). Performance enhancement of MEMS-guided four beam piezoelectric transducers for energy harvesting and acceleration sensing. Int. J. Mod. Phys. B.

[9] Chang J, Wang Q, Zhang X (2009). A fiber bragg grating acceleration sensor interrogated by a DFB laser diode. Laser Phys..

[10] Guo Y, Chen M, Xiong Li (2021). Fiber Bragg grating based acceleration sensors: a review. Sens. Rev..

[11] Chen J, Liu B, Zhang H (2011). Review of fiber Bragg grating sensor technology. Front. Optoelectron. China.

[12] Goto H, Kaneko Y, Young J (2019). Extreme accelerations during earthquakes caused by elastic flapping effect. Sci. Rep..

[13] Zhao X, Jia Z, Fan W (2021). A Fiber Bragg Grating acceleration sensor with temperature compensation. Optik.

[14] Li T, Tan Y, Han X (2017). Diaphragm based fiber Bragg grating acceleration sensor with temperature compensation. Sensors.

[15] Khan MM, Panwar N, Dhawan R (2014). Modified cantilever beam shaped FBG based accelerometer with self temperature compensation. Sens. Actuators A.

[16] Casas-Ramos MA, Sandoval-Romero GE (2017). Fiber optic mechanical vibration sensor. Vibroengineering Procedia.

[17] Wang D, Wu Y, Song Y (2021). A double-mass block acceleration sensor based on FBG. Opt. Fiber Technol..

[18] Wu H, Lin Q, Han F (2020). Design and analysis of high-frequency fiber Bragg grating vibration sensor. Meas. Sci. Technol..

